# Precision Sensorimotor Control in Aging FMR1 Gene Premutation Carriers

**DOI:** 10.3389/fnint.2019.00056

**Published:** 2019-10-02

**Authors:** Walker S. McKinney, Zheng Wang, Shannon Kelly, Pravin Khemani, Su Lui, Stormi P. White, Matthew W. Mosconi

**Affiliations:** ^1^Clinical Child Psychology Program, Life Span Institute and Kansas Center for Autism Research and Training (K-CART), University of Kansas, Lawrence, KS, United States; ^2^Department of Occupational Therapy, University of Florida, Gainesville, FL, United States; ^3^Department of Neurology, Swedish Neuroscience Institute, Seattle, WA, United States; ^4^Department of Radiology, Huaxi Magnetic Resonance Research Center, West China Hospital of Sichuan University, Chengdu, China; ^5^Department of Pediatrics, Marcus Autism Center, Emory University School of Medicine, Atlanta, GA, United States

**Keywords:** fragile X-associated tremor/ataxia syndrome, FMR1 premutation, sensorimotor, precision grip, neurodegeneration, bradykinesia, dysmetria

## Abstract

**Background:**

Individuals with premutation alleles of the *FMR1* gene are at risk of developing fragile X-associated tremor/ataxia syndrome (FXTAS), a neurodegenerative condition affecting sensorimotor function. Information on quantitative symptom traits associated with aging in premutation carriers is needed to clarify neurodegenerative processes contributing to FXTAS.

**Materials and Methods:**

26 *FMR1* premutation carriers ages 44–77 years and 31 age-matched healthy controls completed rapid (2 s) and sustained (8 s) visually guided precision gripping tasks. Individuals pressed at multiple force levels to determine the impact of increasing the difficulty of sensorimotor actions on precision behavior. During initial pressing, reaction time, the rate at which individuals increased their force, the duration of pressing, and force accuracy were measured. During sustained gripping, the complexity of the force time series, force variability, and mean force were examined. During relaxation, the rate at which individuals decreased their force was measured. We also examined the relationships between visuomotor behavior and cytosine-guanine-guanine (CGG) repeat length and clinically rated FXTAS symptoms.

**Results:**

Relative to controls, premutation carriers showed reduced rates of initial force generation during rapid motor actions and longer durations of their initial pressing with their dominant hand. During sustained force, premutation carriers demonstrated reduced force complexity, though this effect was specific to younger premutation carries during dominant hand pressing and was more severe for younger relative to older premutation carriers at low and medium force levels. Increased reaction time and lower sustained force complexity each were associated with greater CGG repeat length for premutation carriers. Increased reaction time and increased sustained force variability were associated with more severe clinically rated FXTAS symptoms.

**Conclusion:**

Overall our findings suggest multiple sensorimotor processes are disrupted in aging premutation carriers, including initial force control guided by feedforward mechanisms and sustained sensorimotor behaviors guided by sensory feedback control processes. Results indicating that sensorimotor issues in aging premutation carriers relate to both greater CGG repeat length and clinically rated FXTAS symptoms suggest that quantitative tests of precision sensorimotor ability may serve as key targets for monitoring FXTAS risk and progression.

## Background

Fragile X syndrome is the most common heritable form of intellectual disability, and it is caused by “full” mutations of the *FMR1* gene consisting of >200 cytosine-guanine-guanine (CGG) repeats (Kremer et al., 1991). Premutations of the *FMR1* gene involving 55–200 CGG repeats also confer risk for multiple subclinical issues as well as medical, psychiatric, and neurodegenerative conditions (Lozano et al., 2014) including fragile X-associated tremor/ataxia syndrome (FXTAS). FXTAS is a neurodegenerative disease in which patients present with a variety of sensorimotor, cognitive, psychiatric and medical issues, as well as cerebellar and cortical degeneration typically beginning at ages 50–70 years (Brunberg et al., 2002; Jacquemont et al., 2003). The defining clinical symptoms of FXTAS include intention tremor, gait ataxia, and Parkinsonism (Hagerman et al., 2001; Jacquemont et al., 2003; Leehey et al., 2007; Juncos et al., 2011), though some patients also demonstrate cognitive decline and psychiatric issues (Grigsby et al., 2008). Pathology of the middle cerebellar peduncle (MCP sign), cerebral atrophy, and intranuclear inclusions also are associated with FXTAS (Brunberg et al., 2002; Greco et al., 2006). Still, symptom presentation is highly variable across patients, and objective, quantitative tools are needed to identify aging premutation carriers most at risk of developing FXTAS, track disease progression, and determine neurobiological mechanisms (Jacquemont et al., 2004; Leehey et al., 2007).

Prior quantitative studies have indicated that premutation carriers with FXTAS and elderly, asymptomatic premutation carriers each show sensorimotor issues. For example, FXTAS patients show increased postural sway relative to healthy aging individuals (Aguilar et al., 2008), while aging premutation carriers with and without FXTAS each show postural sway during standing that is associated with greater CGG repeat length (Kraan et al., 2013; O’Keefe et al., 2015; Wang et al., 2019). Studies of fine motor abilities critical to everyday activities have indicated that asymptomatic *FMR1* premutation carriers (Shickman et al., 2018) and FXTAS patients (Schneider et al., 2012) show reduced motor speed. Park et al. (2019) also reported increased force variability during sustained finger abduction implicating feedback processes involved in reactively adjusting ongoing precision motor behaviors in response to sensory error information. Importantly, Shickman et al. (2018) documented that more severe fine motor issues were associated with greater CGG repeat length in asymptomatic aging premutation carries, suggesting fine motor deficits may covary with FXTAS risk. While these studies indicate tests of fine motor control may be useful for quantifying clinical and subclinical issues in aging premutation carriers, precise and translational measurements that comprehensively assess multiple sensorimotor processes, including the initiation, maintenance, and termination of behavior, are needed to define affected systems, clarify neurobiological mechanisms of FXTAS, and monitor both disease risk and progression.

One candidate approach for characterizing multiple sensorimotor processes in premutation carriers is studying visually guided precision gripping. Precision gripping is important for many daily living activities (e.g., writing, grasping objects), and multiple studies have documented atypical precision gripping behavior in neurodevelopmental (Mosconi et al., 2015; Wang et al., 2015) and neurodegenerative conditions that affects patients’ quality of life (Vaillancourt et al., 2001a, c). Further, the neural bases of visually guided precision gripping have been studied extensively suggesting that clarifying spared and affected processes may help identify key brain mechanisms associated with different clinical conditions (Ehrsson et al., 2000; Kuhtz-Buschbeck et al., 2008; Prodoehl et al., 2009; Neely et al., 2013). During precision gripping, individuals initiate a “rise phase” in which they rapidly increase their force output to reach a target level. Due to afferent delays of sensory feedback information, initial pressing is guided by internal action plans and often results in initial dysmetria (e.g., overshooting at lower force levels; undershooting at higher force levels), especially during rapid compared to longer duration actions (Desmurget et al., 1999; Potter et al., 2006; Wang et al., 2015). During a subsequent “sustained phase,” individuals reactively adjust motor output to match their target and maintain a more constant level of force integrating feedforward and sensory feedback processes. Increases in sustained force variability and reductions in force complexity each implicate failures in the ability to dynamically and reactively adjust precision motor output in response to sensory feedback (Vaillancourt et al., 2001b; Chu and Sanger, 2009). At the end of precision gripping actions, participants engage in a “relaxation phase” in which they rapidly release their grip force by terminating motor unit firing within agonist muscles supporting gripping (e.g., first dorsal interosseus) and initiating antagonist motor unit firing.

In the present study, we systematically assessed rise, sustained, and relaxation phases of visually guided precision gripping in *FMR1* premutation carriers ages 44–77 years. Our primary goal was to comprehensively characterize precision sensorimotor behaviors in aging *FMR1* premutation carriers as the extent to which initial, sustained, and relaxation phase behaviors are impacted has not yet been assessed. Both rapid and sustained actions were tested in order to determine the differential impact of *FMR1* premutations on sensorimotor feedforward and feedback processes. During rapid sensorimotor tasks, greater demands are placed on feedforward systems responsible for the accuracy and rapid execution of initial motor plans (Ghez et al., 1991). During sustained sensorimotor action, the integrity of sensorimotor feedback processes responsible for the online translation of sensory error information into corrective motor action is tested (Desmurget and Grafton, 2000; Vaillancourt et al., 2003). We also assessed sensorimotor behavior across multiple force levels, allowing us to assess the effect of increased task requirements on precision sensorimotor behavior. By examining a large age range, we were able to determine whether visually guided precision gripping issues were more prominent at relatively earlier stages of aging suggesting that they may be prodromal markers of degeneration, or whether they may become more prominent later suggesting decline at advanced ages. Gripping was tested across both hands to determine if neurodegenerative processes associated with aging in *FMR1* premutation carriers may be lateralized as previously suggested (Przybyla et al., 2011; Raw et al., 2012). We also examined the relationship between sensorimotor outcomes, FXTAS clinical symptoms, and CGG repeat length to determine the utility of our measures for characterizing sensorimotor deterioration associated with the severity of and risk for FXTAS.

## Materials and Methods

### Participants

Twenty-six premutation carriers and 31 healthy controls completed sensorimotor testing (Table 1). Controls and carriers did not differ on age, sex ratio, or handedness. No premutation carriers had an existing diagnosis of any neurological disorder, nor did they self-report any motor (e.g., gait ataxia, intention tremor) or memory issues. Controls were excluded for current or past neurodegenerative, neurological, or major psychiatric disorders (e.g., schizophrenia, bipolar disorder). Controls also were excluded for a family history of fragile X syndrome or intellectual/developmental disabilities in first- or second-degree relatives. Participants were excluded if they reported any neurological or musculoskeletal disorder that could cause atypical sensorimotor functioning or a history of medications known to affect sensorimotor functioning, including antipsychotics, stimulants, or benzodiazepines (Reilly et al., 2008).

**TABLE 1:** Demographic and clinical characteristics.

	**Controls (*n* = 31)**	**Premutation carriers (*n* = 26)**	***t***	***p***
Age (years)	53 (10)	57 (9)	−1.39	0.169
Age range (years)	40–73	44–77	–	–
Sex (% male)^†^	39%	23%	1.60^†^	0.206
Handedness (% right)^†^	90%	96%	0.74^†^	0.391
FSIQ	109 (13)	99 (12)	2.93	0.005^∗∗^
Dominant hand MVC	87 (28)	77 (23)	1.42	0.161
Non-dominant hand MVC	84 (32)	82 (27)	0.24	0.811
ICARS total score	–	5 (5)	–	–
ICARS total range	–	0–19	–	–
CGG repeat length	–	82 (17)	–	–
CGG repeat length range	–	55–110	–	–

*FSIQ: full-scale IQ; ICARS: International Cooperative Ataxia Rating Scale; CGG: cytosine-guanine-guanine. Variables are presented as: mean (SD); ^∗∗^*p* < 0.01; ^†^chi-square statistic; a group by hand interaction of MVC is reported in Table 2.*

*FMR1* premutation carriers were identified through local fragile X clinics and postings on local and national fragile X association LISTSERVs. Control participants were recruited through community advertisements. This study was carried out in accordance with the recommendations of and was approved by the University of Texas Southwestern Medical Center Institutional Review Board. All subjects provided written informed consent after a complete description of the study in accordance with the Declaration of Helsinki.

### Neurological Evaluations

*FMR1* premutation carriers completed a clinical exam by a neurologist with expertise in movement control in aging (PK). The clinical exam included administration of the International Cooperative Ataxia Rating Scale (Trouillas et al., 1997). The ICARS is comprised of 19 sections examining postural and gait disturbances, ataxia, dysarthria, and oculomotor behavior. Higher scores indicate more severe neuromotor issues. The ICARS has been validated previously for diagnosis of ataxia in patients with focal cerebellar lesions (Schoch et al., 2007), hereditary spinocerebellar and Friedrich’s ataxia (Schmitz-Hubsch et al., 2006). Nine premutation carriers did not complete the clinical evaluation due to scheduling difficulties. For the 17 premutation carriers who completed the clinical visit, ICARS scores are presented in Table 1.

### Sensorimotor Testing

**FIGURE 1:**
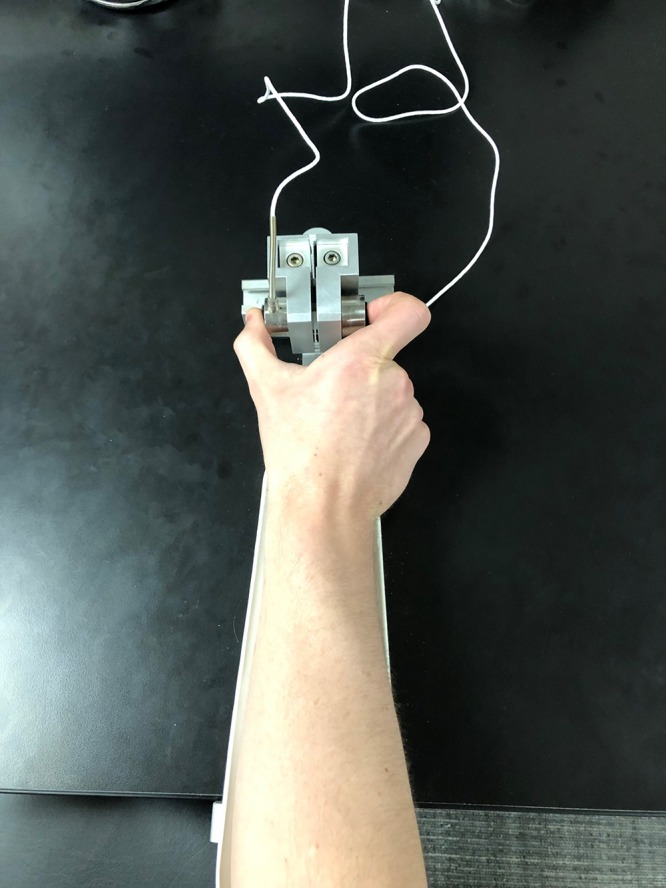
The custom-made arm brace and load cells for precision grip testing. Participants pressed with their thumb and forefinger against two precision load cells while viewing two horizontal bars displayed vertically on the screen.

Participants completed two tests of sensorimotor behavior differentiated by the trial duration and inter-trial interval (“rapid” trials included 2 s of pressing alternating with 2 s of rest, and “sustained” trials included 8 s of pressing alternating with 8 s of rest). For both tests, stimuli were presented on a 102 cm (40 inches) Samsung LCD monitor with a resolution of 1366 × 768 and a 120 Hz refresh rate. Participants were tested in a darkened room while seated 52 cm from the display monitor with their elbow at 90° and their forearm resting in a relaxed position on a custom-made arm brace. The arm brace was clamped to a table to keep the participant’s arm position stable throughout testing (Figure 1). The participant’s hand was pronated and lay flat with digits comfortably extended. Participants used their thumb and index finger to press against two opposing precision load cells (ELFF-B4-100N; Entran) 1.27 cm in diameter secured to a custom grip device attached to the arm brace. A Coulbourn (V72-25) resistive bridge strain amplifier received analog signal from the load cells. Data were sampled at 200 Hz with a 16-bit analog-to-digital converter (DI-720; DATAQ Instruments) and converted to Newton of force using a calibration factor derived from known weights before the study (Mosconi et al., 2015).

### Procedures

Before testing, each participant’s maximum voluntary contraction (MVC) was calculated separately for each hand using the average of the maximum force output during three trials in which participants pressed as hard as they could for 3 s.

**FIGURE 2:**
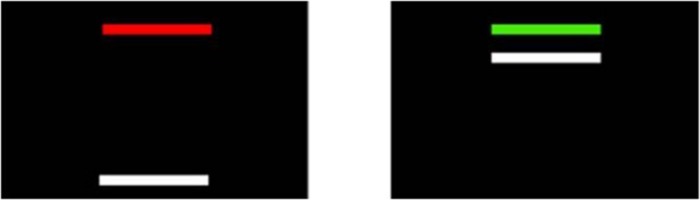
Sensorimotor test stimuli. Participants pressed when the red bar turned green in order to move the white bar up to the target green bar. They were instructed to maintain their force level at the level of the green bar as steadily as possible.

During sensorimotor testing, participants viewed a horizontal white force bar that moved upward with increased force and downward with decreased force and a static target bar that was red during rest and turned green to cue the participant to begin pressing at the beginning of each trial (Figure 2). Participants received two instructions: (1) press the load cells as quickly as possible when the red target bar turns green, and (2) keep pressing so that the force bar stays as steady as possible at the level of the green target bar. These instructions were identical for the two versions of the task described below.

“Rapid” (2 s) and “sustained” (8 s) trials were administered at 15, 45, and 85% of each individual’s MVC. During the rapid test, two blocks of five trials were presented for each hand at each force level (2 hands × 3 force levels × 2 blocks × 5 trials = 60 rapid trials). Each 2 s rapid trial alternated with 2 s rest periods. A 15 s rest block was provided after each block of trials. During the sustained test, participants completed two blocks of three trials for each hand at each force level (2 hands × 3 force levels × 2 blocks × 3 trials = 36 sustained trials). Eight seconds trials were followed by 8 s rest periods, and each block was separated by 15 s of rest. For both tests, the same hand was never tested on consecutive blocks. The order of force levels was randomized across blocks. The order of the two experiments was randomly assigned to each participant. Participants self-reported their dominant hand.

### Sensorimotor Data Processing

Force traces for each trial were low-pass filtered via a double-pass 4th-order Butterworth filter at a cutoff of 15 Hz in MATLAB. Data were analyzed using custom MATLAB scripts previously developed by our lab (Wang et al., 2015).

Data from three distinct phases were analyzed. During the initial *rise* phase in which individuals pressed on the load cells to reach the target level, we examined reaction time, peak rate of force increase (i.e., the maximum value of the first derivative of the force trace), the duration of the period in which individuals increased their force, and the accuracy of their initial force output. The onset of the rise phase was calculated as the time at which the rate of force increase first exceeded 5% of the peak rate of force increase and remained above this level for at least 100 ms (Grafton and Tunik, 2011; Wang et al., 2015). Reaction time was calculated as the difference between rise phase onset and the appearance of the start cue. The rise phase offset was calculated as the time-point when the rate of force increase fell below 5% of the peak rate of force increase, and the force level was within 90–110% of the mean force of the sustained phase (Wang et al., 2015). The peak rate of force increase was defined as the maximum value of the first derivative of the force trace. Rise phase duration was then calculated as the difference between the rise phase offset and rise phase onset. Rate of force increase and duration of initial force output were analyzed relative to rise phase force output to account for differences in force kinetics attributable to differences in force amplitude. Force accuracy for the rise phase was calculated as the force at rise offset divided by the target force (i.e., $\mathrm{Rise}\,\mathrm{Accuracy}=\frac{\left(F_{\text{rise}}\right)}{\left(F_{\text{target}}\right)}$). Values below 1 represent an undershooting of the target force and values above 1 reflect overshooting of the target force. An accuracy score of 1 indicates perfect accuracy. The entire rise phase was excluded if participants began gripping before the start cue, or if they returned to baseline prior to reaching 90% of the target force. Rise phase data for both the rapid and sustained tasks were analyzed within the same model to allow for the analysis of task effects (i.e., rapid vs. sustained). Consistent with prior studies, participants were expected to show faster reaction times, more rapid increases in force, shorter rise phase duration and reduced accuracy during rapid compared to sustained actions (Wang et al., 2015).

To determine the extent to which participants could maintain a constant level of force using visual feedback, the *sustained* phase was examined and defined as the period following rise phase offset and prior to the appearance of the stop cue. Due to the brief duration of rapid trials, the sustained phase was only examined during 8 s trials. The mean force of the time series was calculated to determine individuals’ ability to complete the task. The variability of the force time series was calculated using the following procedures: first, force data were linearly detrended to account for systematic changes in mean force over the course of the trial (e.g., data drift). Second, the within-trial standard deviation (SD) of the force time series was calculated. To examine the time-dependent structure of the time series, the approximate entropy (ApEn) was calculated for each trial (Slifkin and Newell, 1999; Vaillancourt et al., 2001b). ApEn returns a value between 0 and 2, reflecting the predictability of future values in a time series based on previous values. For example, a sine wave has accurate short- and long-term predictability, corresponding to an ApEn value near 0. High irregularity of the data, reflective of the independence of each force value, returns an ApEn near 2. The algorithm and parameter settings for these calculations (*m* = 2; *r* = 0.2 × SD of the signal) were identical to previous work (Vaillancourt and Newell, 2000). Sustained phase variables were excluded if fewer than 4 s of data were available or if participants returned to baseline for more than 1 s (e.g., a > 1 s dip of the force signal).

In order to determine the rate at which individuals released force at the end of trials, the *relaxation* phase also was examined. The onset of the relaxation phase was defined as the first point following the stop cue (target bar turned red) at which velocity (i.e., rate of change of force) went below 5% of the peak velocity and remained at that level or below for at least 100 ms. The offset of the relaxation phase was defined as the first point at which velocity rose back above 5% of the peak relaxation velocity. We examined the rate of force decrease during the relaxation phase. The peak rate of force decrease was identified as the minimum value of the first derivative of the force trace following the stop cue. To control for differences in force level prior to force release, the rate of force decrease was examined relative to force amplitude prior to relaxation. Rate of force relaxation was not examined if the participant released force prior to the stop cue. Relaxation phase data was examined for both the rapid and sustained tasks in the same model.

### CGG Repeat Count

All premutation carriers provided blood samples to confirm premutation status. *FMR1* CGG repeat count was quantified using molecular testing conducted at Dr. Elizabeth Berry-Kravis’ Molecular Diagnostic Laboratory at Rush University. Genomic DNA was isolated from peripheral blood leukocytes samples. The *FMR1* polymerase chain reaction (PCR) test with quantification of allele-specific CGG repeat count was performed using commercially available kits (Asuragen, Inc., Austin, TX, United States). For women, CGG repeat analyses reflect the longest CGG repeat of the two alleles.

### Cognitive Measures

Cognitive functioning was assessed using the abbreviated battery of the Stanford-Binet Intelligence Scales, Fifth Edition (SB-5) including non-verbal fluid reasoning and verbal knowledge sub-sections (Roid, 2003). One participant did not complete the SB-5 because they were not fluent in English. Healthy controls had significantly higher full-scale IQs (*M* = 109.3, *SD* = 12.8) than premutation carriers (*M* = 99.5, *SD* = 12.1), *t*(54) = 2.93, *p* < 0.01, though IQ was in the average range for both groups (Table 1).

### Statistical Analyses

To determine whether sensorimotor ability differed according to premutation carrier status, linear multilevel mixed effect (MLM) analyses were conducted (Bates et al., 2015; Koller, 2016). This approach allows for the simultaneous analysis of within- and between-subject fixed effects while allowing within-subject factors to differ for each participant as random effects. This approach also allows for the analysis of interactions within the repeated measures design including participants with missing data (e.g., failed to complete dominant hand trials at 85% MVC) without listwise deletion of that participant. Task (rapid vs. sustained) and condition effects (percent MVC, hand) were identified as level 1 predictors and subject effects (group, age) were identified as level 2 predictors. Random variance components for the intercept (subject) also were analyzed. To maintain relatively parsimonious models, five-way interactions were not analyzed. Initial models included all two-, three-, and four-way interactions, after which variables and interactions were removed and model fit was compared between the previous and current models using a likelihood ratio test. Only variables which significantly (*p* < 0.05) improved model fit were incorporated into final models. Non-normally distributed variables were log-transformed. Final models used robust linear mixed effect modeling to provide more stringent fixed effect estimates and standard errors while reducing the impact of outliers (Pinheiro et al., 2001). Due to concerns with Type 1 error when interpreting robust estimates with traditional *p*-value cut-offs, we followed best practice guidelines and significant results are reported if |*t|* ≥ 1.96 (Luke, 2017). Age was centered for all models, and each categorical predictor was dummy coded with the following conditions serving as baseline references: healthy controls, 15% MVC, dominant hand, rapid (2 s) task. Based on these references, the intercept for each model was interpreted as the predicted value of the dependent outcome for an average aged (54.87 years) healthy control during the 15% MVC dominant hand rapid task. Predicted values are then obtained by adding the relevant fixed effect and interaction estimates. Main effect and interaction effect results are reported relative to baseline reference values. Due to the MVC manipulation having three levels, MVC percent main and interaction effects are presented separately for 45 and 85% MVC relative to the 15% MVC reference condition. Significant task and age effects are reported followed by group and group interaction effects. Mixed effect modeling was conducted using the *robustlmm* and *lmer* packages within R version 3.6.0 (Bates et al., 2015; Koller, 2016).

Due to the non-normal distribution of CGG repeat length and ICARS scores, the relationships between sensorimotor outcomes, ICARS scores, and CGG repeat length were examined using Spearman’s rank-order correlations. Linear regression was used to determine if total ICARS scores were related to age, CGG repeat length, or the interaction of age and CGG repeat length. Due to the large number of correlations that were performed, only results with *p* < 0.01 were interpreted as significant. Correlational and regression analyses were conducted using IBM SPSS Statistics 25.

## Results

### Maximum Voluntary Contraction

**FIGURE 3:**
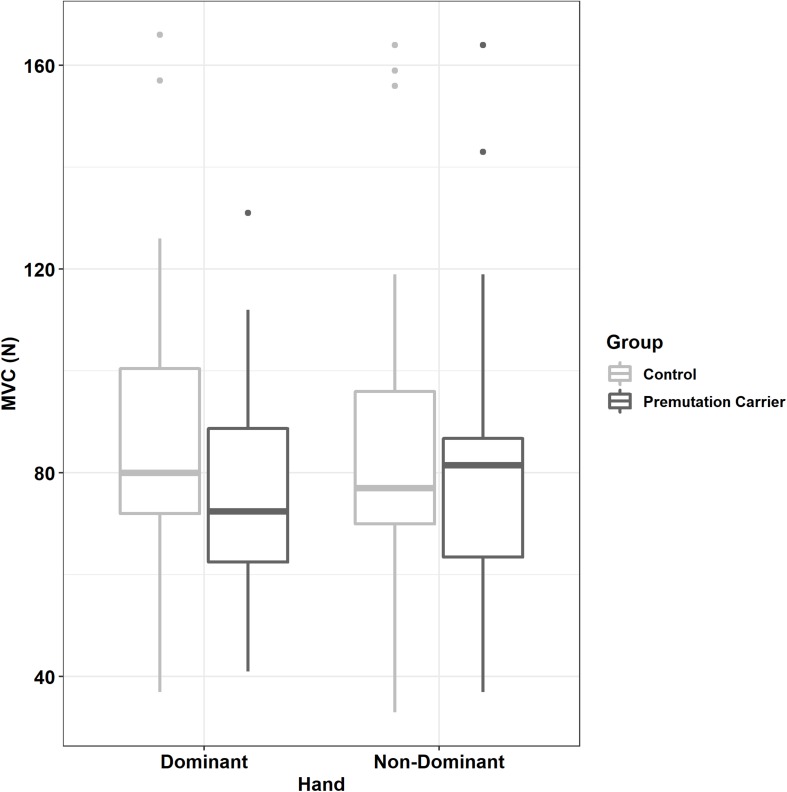
Maximum voluntary contract (MVC) as a function of group and hand. Relative to controls, premutation carriers showed a greater difference between their dominant and non-dominant hand MVC.

Relative to controls, premutation carriers showed a greater difference between their dominant and non-dominant hand MVC (Figure 3 and Tables 1, 2; group × hand: β = 7.16, *SE* = 3.47, *p* = 0.039, partial *R*^2^ = 0.010). MVC was not related to age (β = −2.01, *SE* = 4.00, *p* = 0.616, partial *R*^2^ = 0.029), and the relationship between age and MVC did not differ between groups (group × age: β = 6.47, *SE* = 6.28, *p* = 0.303, partial *R*^2^ = 0.055).

**TABLE 2:** Best fitting multilevel models for subject MVC.

	**Fixed effect estimates**	**Estimate (SE)**	***t***	**Partial*R*^2^**
MVC	Level 1 variables			
	Intercept	83.93 (4.20)	19.96^∗^	−
	Hand	−3.59 (2.34)	–1.53	0.003
	Level 2 variables			
	Group	−8.30 (6.23)	–1.33	0.031
	Interaction variables			
	Group × hand	7.16 (3.46)	2.07^∗^	0.010
	
	**Random effect variances**	**Variance (SD)**		
	
	Level 1 residual (ε_it_)	80.68 (8.98)	−	–
	Level 2 intercept (μ_0i_)	440.24 (20.98)	−	–

*^∗^*p* < 0.05 using the *t*-as-*z* approach; MVC: maximum voluntary contraction; partial *R*^2^ reflects the proportion of residual variation accounted for by the fixed effect when added to the same model without the fixed effect.*

### Rise Phase

#### Reaction Time

Participants showed shorter reaction times during the rapid task relative to the sustained task (Table 3; β = 0.14, *SE* = 0.02, *p* < 0.001, partial *R*^2^ = 0.050). Reaction time increased with increases in target MVC percent (15% vs. 45% MVC: β = 0.10, *SE* = 0.02, *p* < 0.001, partial *R*^2^ = 0.015; 15% vs. 85% MVC: β = 0.17, *SE* = 0.02, *p* < 0.001, partial *R*^2^ = 0.036) and age (β = 0.10, *SE* = 0.04, *p* = 0.014, partial *R*^2^ = 0.074).

**TABLE 3:** Best fitting multilevel models for reaction time and rise phase rate of force increase.

	**Fixed effect estimates**	**Estimate (SE)**	***t***	**Partial *R*^2^**
Reaction time	Level 1 variables			
	Intercept	−1.08(0.04)	−24.01^∗^	–
	15% vs. 45% MVC	0.10 (0.02)	4.62^∗^	0.015
	15% vs. 85% MVC	0.17 (0.02)	7.65^∗^	0.036
	Task	0.14 (0.02)	7.97^∗^	0.050
	Level 2 variables			
	Age	0.10 (0.04)	2.47^∗^	0.074
	
	**Random effect variances**	**Variance (SD)**		
	
	Level 1 residual (ε_it_)	0.05 (0.22)	−	–
	Level 2 intercept (μ_0i_)	0.09 (0.30)	−	–

	**Fixed effect estimates**	**Estimate (SE)**	***t***	**Partial *R*^2^**

Rate of force increase	Level 1 variables			
	Intercept (15% MVC)	1.70 (0.05)	31.53^∗^	–
	15% vs. 45% MVC	−0.22(0.04)	−6.03^∗^	0.050
	15% vs. 85% MVC	−0.34(0.04)	−9.14^∗^	0.104
	Hand	0.10 (0.02)	4.43^∗^	0.025
	Task	−0.16(0.04)	−3.79^∗^	0.015
	Level 2 variables			
	Group	−0.13(0.07)	–1.86	0.025
	Age	−0.07(0.03)	−2.03^∗^	0.049
	Interaction variables			
	Group × task	0.13 (0.04)	2.91^∗^	0.010
	15% vs. 45% MVC × task	−0.11(0.05)	−2.17^∗^	0.008
	15% vs. 85% MVC × task	−0.13(0.05)	−2.39^∗^	0.009
	
	**Random effect variances**	**Variance (SD)**		
	
	Level 1 residual (ε_it_)	0.07 (0.27)	−	–
	Level 2 intercept (μ_0i_)	0.05 (0.23)	−	–

*^∗^*p* < 0.05 using the *t*-as-*z* approach; MVC: maximum voluntary contraction; partial *R*^2^ reflects the proportion of residual variation accounted for by the fixed effect when added to the same model without the fixed effect.*

No significant group differences or group interactions were identified for reaction time.

#### Rate of Force Increase

Participants demonstrated a higher rate of force increase during the rapid compared to the sustained task (Table 3; β = −0.16, *SE* = 0.04, *p* < 0.001, partial *R*^2^ = 0.015). After controlling for target amplitude (i.e., force level at the end of the rise phase), rate of force increase also was reduced at higher compared to lower MVC target levels (15% vs. 45% MVC: β = −0.22, *SE* = 0.04, *p* < 0.001, partial *R*^2^ = 0.050; 15% vs. 85% MVC: β = −0.34, *SE* = 0.04, *p* < 0.001, partial *R*^2^ = 0.104). Rate of force increase was greater with the non-dominant compared to the dominant hand (β = 0.10, *SE* = 0.02, *p* < 0.001, partial *R*^2^ = 0.025) and slowed with age (β = −0.07, *SE* = 0.03, *p* = 0.042, partial *R*^2^ = 0.049).

**FIGURE 4:**
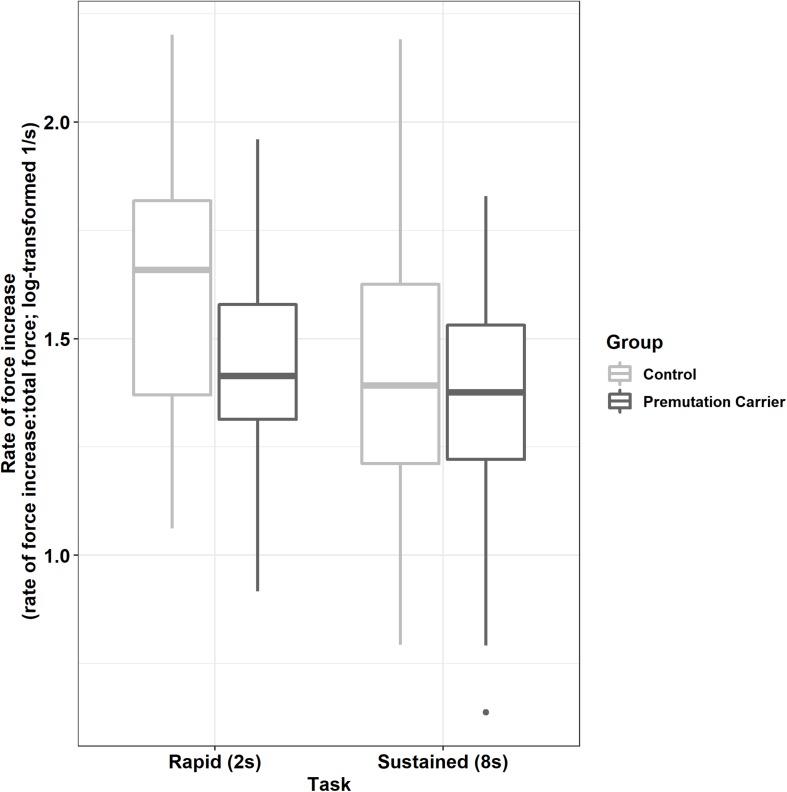
Peak rate of force increase (relative to initial force output) as a function of group and task. Relative to controls, premutation carriers show a reduced rate of force increase during rapid pressing.

Premutation carriers showed a reduced rate of force increase relative to controls during the rapid but not the sustained task (Figure 4; group × task: β = 0.13, *SE* = 0.04, *p* = 0.004, partial *R*^2^ = 0.009).

#### Rise Phase Duration

For all participants, rise phase duration was greater during the rapid compared to the sustained task (Table 4; β = −0.31, *SE* = 0.02, *p* < 0.001, partial *R*^2^ = 0.214) and was increased at higher compared to lower MVC target levels (15% vs. 45% MVC: β = −1.04, *SE* = 0.02, *p* < 0.001, partial *R*^2^ = 0.680; 15% vs. 85% MVC: β = −1.59, *SE* = 0.02, *p* < 0.001, partial *R*^2^ = 0.831).

**TABLE 4:** Best fitting multilevel models for rise phase duration and accuracy.

	**Fixed effect estimates**	**Estimate (SE)**	***t***	**Partial *R*^2^**
Rise phase duration	Level 1 variables			
	Intercept	−2.43(0.06)	−38.86^∗^	−
	15% vs. 45% MVC	−1.04(0.02)	−46.72^∗^	0.680
	15% vs. 85% MVC	−1.59(0.02)	−71.98^∗^	0.831
	Hand	0.04 (0.02)	1.64	0.002
	Task	−0.31(0.02)	−17.11^∗^	0.214
	Level 2 variables			
	Group	0.15 (0.09)	1.62	0.025
	Interaction variables			
	Group × hand	−0.12(0.04)	−3.28^∗^	0.011
	
	**Random effect variances**	**Variance (SD)**		
	
	Level 1 residual (ε_it_)	0.05 (0.23)	−	–
	Level 2 intercept (μ_0i_)	0.10 (0.32)	−	–

	**Fixed effect estimates**	**Estimate (SE)**	***t***	**Partial*R*^2^**

Rise accuracy	Level 1 variables			
	Intercept (15% MVC)	1.04(0.45×10^−2^)	232.14^∗^	–
	15% vs. 45% MVC	−0.04(0.01)	−7.22^∗^	0.076
	15% vs. 85% MVC	−0.07(0.01)	−12.66^∗^	0.144
	Task	−0.02(0.01)	−3.29^∗^	0.039
	Interaction variables			
	15% vs. 45% MVC × task	0.01 (0.01)	1.44	0.016
	15% vs. 85% MVC × task	0.02 (0.01)	1.86	0.017
	
	**Random effect variances**	**Variance (SD)**		
	
	Level 1 residual (ε_it_)	0.18 × 10^−2^ (0.04)	−	–
	Level 2 intercept (μ_0i_)	0.01 × 10^−2^ (0.01)	−	–

*^∗^*p* < 0.05 using the *t*-as-*z* approach; MVC: maximum voluntary contraction; Partial *R*^2^ reflects the proportion of residual variation accounted for by the fixed effect when added to the same model without the fixed effect.*

**FIGURE 5:**
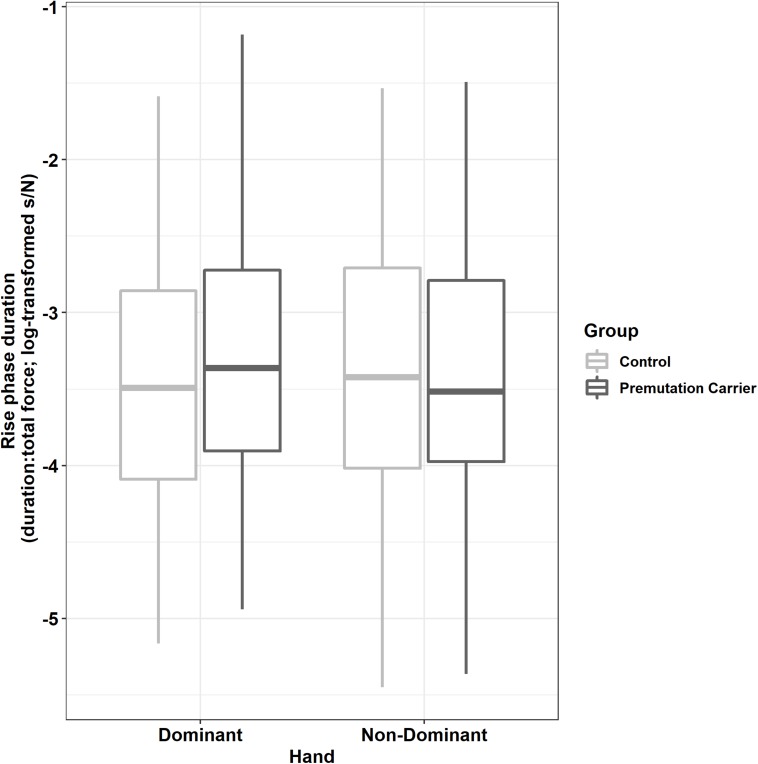
Rise phase duration (relative to initial force output) as a function of group and hand. Relative to controls, premutation carriers showed an increased time to reach target force levels when using their dominant hand but not their non-dominant hand.

Relative to controls, premutation carriers showed longer rise phase durations, but only for their dominant hand (Figure 5; group × hand: β = −0.12, *SE* = 0.04, *p* = 0.001, partial *R*^2^ = 0.011).

#### Rise Phase Accuracy

Across tasks, participants overshot target force levels at 15% MVC (*M* = 1.05; *SD* = 0.14), showed greater accuracy at 45% MVC (*M* = 0.99; *SD* = 0.04), and then undershot target force level at 85% MVC (*M* = 0.96; *SD* = 0.05). During the rapid task, participants demonstrated greater levels of overshooting compared to the sustained task at 15% MVC (β = −0.02, *SE* = 0.01, *p* = 0.001, partial *R*^2^ = 0.039) but similar accuracy at 45% (15% vs. 45% MVC × task: β = 0.01, *SE* = 0.01, *p* = 0.150, partial *R*^2^ = 0.016) and 85% MVC (15% vs. 85% MVC × task: β = 0.02, *SE* = 0.01, *p* = 0.062, partial *R*^2^ = 0.017).

There were no significant group differences or group interactions for rise phase accuracy.

### Sustained Phase

#### ApEn

Participants demonstrated reduced ApEn at higher compared to lower target force levels (Table 5; 15% vs. 45% MVC: β = −0.06, *SE* = 0.02, *p* < 0.001, partial *R*^2^ = 0.136; 15% vs. 85% MVC: β = −0.13, *SE* = 0.02, *p* < 0.001, partial *R*^2^ = 0.026).

**TABLE 5:** Best fitting multilevel models for sustained phase variables.

	**Fixed effect estimates**	**Estimate (SE)**	***t***	**Partial *R*^2^**
ApEn	Level 1 variables			
	Intercept	0.51 (0.02)	30.06^∗^	–
	15% vs. 45% MVC	−0.05(0.02)	−3.63^∗^	0.136
	15% vs. 85% MVC	−0.13(0.02)	−8.47^∗^	0.026
	Hand	−0.01(0.01)	–0.51	0.001
	Level 2 variables			
	Group	−0.03(0.03)	–1.22	0.004
	Age	−0.01(0.02)	–0.74	0.002
	Interaction variables			
	Group × 15% vs. 45% MVC	0.03×10^−2^(0.02)	0.01	<0.001
	Group × 15% vs. 85% MVC	0.03 (0.02)	1.28	0.002
	Group × hand	−0.01(0.02)	–0.46	0.001
	Group × age	0.05 (0.03)	1.95	0.014
	15% vs. 45% MVC × age	−0.01(0.01)	–0.90	0.002
	15% vs. 85% MVC × age	0.02 (0.01)	1.36	0.003
	Hand × age	0.03 (0.01)	2.26^∗^	0.011
	Group × hand × age	−0.05(0.02)	−2.54^∗^	0.010
	Group × age × 15% vs. 45% MVC	−0.12×10^−2^(0.02)	–0.05	<0.001
	Group × age × 15% vs. 85% MVC	−0.06(0.02)	−2.67^∗^	0.010
	
	**Random effect variances**	**Variance (SD)**		
	
	Level 1 residual (ε_it_)	0.01 (0.08)	−	–
	Level 2 intercept (μ_0i_)	0.38×10^−2^(0.06)	−	–

	**Fixed effect estimates**	**Estimate (SE)**	***t***	**Partial *R*^2^**

Force SD	Level 1 Variables			
	Intercept (15% MVC)	−1.17(0.06)	−18.46^∗^	–
	15% vs. 45% MVC	0.09 (0.06)	15.55^∗^	0.250
	15% vs. 85% MVC	2.00 (0.06)	33.71^∗^	0.631
	
	**Random effect variances**	**Variance (SD)**		
	
	Level 1 residual (ε_it_)	0.01 (0.08)	−	–
	Level 2 intercept (μ_0i_)	0.33×10^−2^(0.06)	−	–

	**Fixed effect estimates**	**Estimate (SE)**	***t***	**Partial *R*^2^**

Mean force	Level 1 variables			
	Intercept	2.53 (0.05)	47.52^∗^	-
	15% vs. 45% MVC	1.07 (0.01)	83.75^∗^	0.677
	15% vs. 85% MVC	1.68 (0.01)	131.09^∗^	0.840
	Hand	−0.05(0.01)	−3.41^∗^	0.004
	Level 2 variables			
	Group	−0.10(0.08)	–1.28	0.015
	Interaction variables			
	Group × hand	0.08 (0.02)	3.91^∗^	0.007
	
	**Random effect variances**	**Variance (SD)**		
	
	Level 1 residual (ε_it_)	0.01 (0.28)	−	–
	Level 2 intercept (μ_0i_)	0.08 (0.28)	−	–

*^∗^*p* < 0.05 using the *t*-as-*z* approach; MVC: maximum voluntary contraction; Partial *R*^2^ reflects the proportion of residual variation accounted for by the fixed effect when added to the same model without the fixed effect.*

**FIGURE 6:**
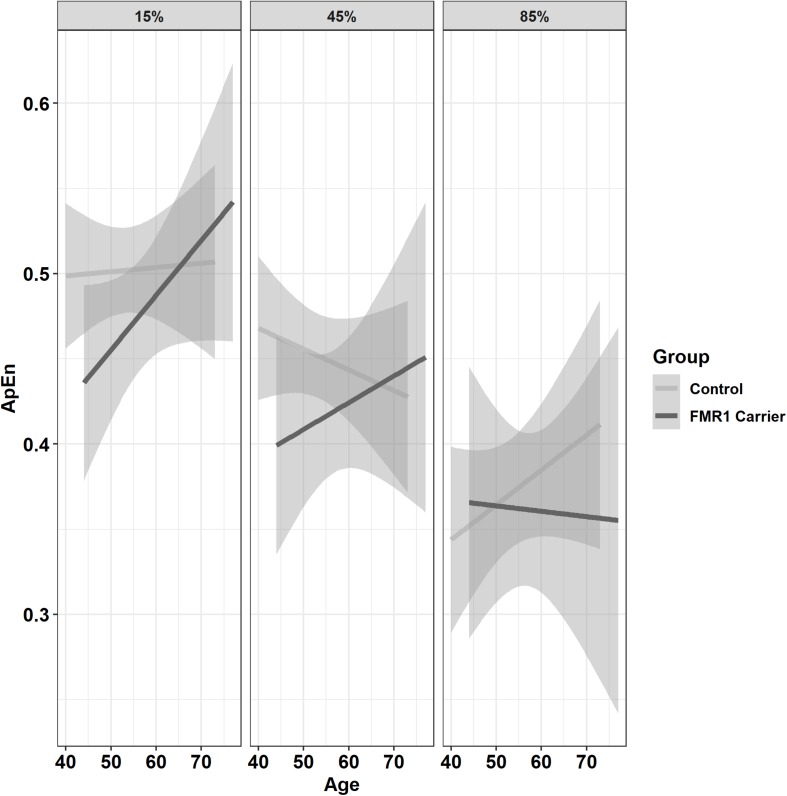
Approximate entropy (ApEn; i.e., force complexity) as a function of group,% MVC, and age (linear fit with 95% confidence intervals). During the 15% and 45% MVC conditions, younger premutation carriers demonstrated reduced force complexity relative to controls, while premutation carriers and controls showed similar levels of force complexity across age at 85% MVC.

**FIGURE 7:**
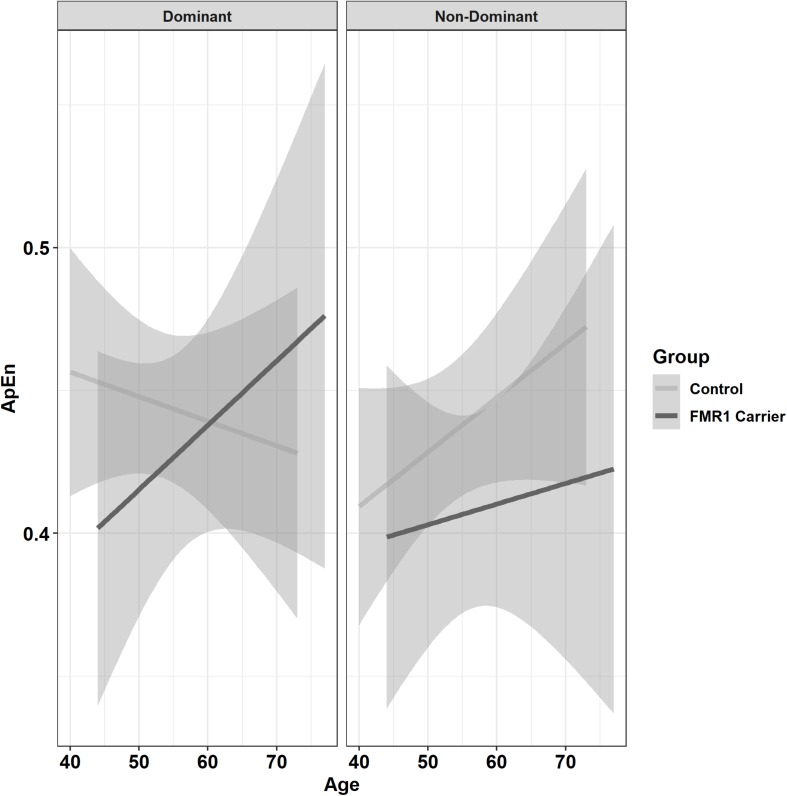
Approximate entropy (ApEn; i.e., force complexity) as a function of group,% MVC, and age (linear fit with 95% confidence intervals). Younger premutation carriers demonstrated reduced force complexity compared to controls when using their non-dominant hand, but premutation carriers only showed reduced force complexity compared to controls at younger but not older ages when using their non-dominant hand.

Premutation carriers showed reduced ApEn relative to controls, and this group difference varied as a function of age and target force level (Figure 6; group × age × 15% vs. 45% MVC: β = −0.001, *SE* = 0.0, *p* = 0.960, partial *R*^2^ < 0.001; group × age × 15% vs. 85% MVC: β = −0.06, *SE* = 0.02, *p* = 0.008, partial *R*^2^ = 0.010). Reduced ApEn in premutation carriers relative to controls was more severe at younger ages during the 15 and 45% MVC conditions but not for the 85% MVC condition. Premutation carriers also showed reduced ApEn that varied as a function of age and hand (Figure 7; group × hand × age: β = −0.05, *SE* = 0.02, *p* = 0.011, partial *R*^2^ = 0.010). Specifically, premutation carriers showed reduced ApEn across age for the non-dominant hand, but this effect was more severe at younger ages relative to older ages for the dominant hand.

#### Force SD

Force SD scaled with target MVC level (Table 5; 15% vs. 45% MVC: β = 0.09, *SE* = 0.06, *p* < 0.001, partial *R*^2^ = 0.250; 15% vs. 85% MVC: β = 2.00, *SE* = 0.06, *p* < 0.001, partial *R*^2^ = 0.631).

There were no significant group differences or group interactions for force SD.

#### Mean Force

Mean sustained force scaled with target MVC level (Table 5; 15% vs. 45% MVC: β = 1.08, *SE* = 0.01, *p* < 0.001, partial *R*^2^ = 0.677; 15% vs. 85% MVC: β = 1.68, *SE* = 0.01, *p* < 0.001, partial *R*^2^ = 0.840) and was reduced in the non-dominant relative to the dominant hand (β = −0.05, *SE* = 0.01, *p* < 0.001, partial *R*^2^ = 0.004).

Compared to controls, premutation carriers demonstrated lower mean force with their dominant hand only (group × hand: β = 0.08, *SE* = 0.02, *p* < 0.001, partial *R*^2^ = 0.007).

### Relaxation Phase

#### Rate of Force Decrease

During the relaxation phase, participants decreased their force level more slowly during higher relative to lower target force levels (15% vs. 45% MVC: β = −0.10, *SE* = 0.01, *p* < 0.001, partial *R*^2^ = 0.079; 15% vs. 85% MVC: β = −0.19, *SE* = 0.01, *p* < 0.001, partial *R*^2^ = 0.219) and during rapid compared to sustained force trials (β = −0.071, *SE* = 0.01, *p* < 0.001, partial *R*^2^ = 0.065).

There were no significant group differences or group interactions for rate of force decrease.

### Sensorimotor Behavior and Clinical/Demographic Outcomes

#### Age

Increased age was significantly associated with more severe ICARS rated FXTAS symptoms (*F*(1,15) = 9.858, *p* = 0.007, *R*^2^ = 0.397). CGG repeat length was not associated with FXTAS symptoms (*F*Δ(1,14) = 1.891, *p* = 0.191, *R*^2^Δ = 0.072).

#### CGG Repeat Length

**FIGURE 8:**
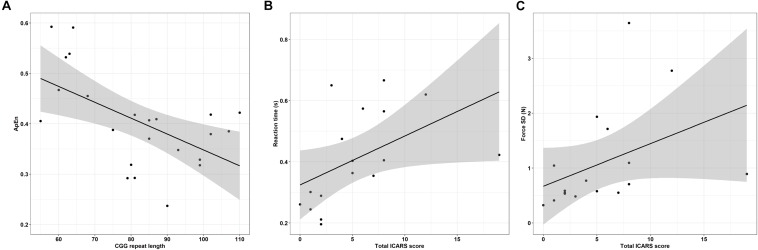
**(A)** CGG repeat length is associated with reduced dominant hand approximate entropy (ApEn) at 45% MVC. Error bars represent 95% CI of a linear fit. **(B)** Increased ICARS rated FXTAS symptoms are associated with longer reaction times (data shown is from rapid test dominant hand trials at 45% MVC). Error bars represent 95% CI of a linear fit. **(C)** Increased ICARS rated FXTAS symptoms are associated with increased force variability (force SD) during the non-dominant hand 45% MVC condition. Error bars represent 95% CI of a linear fit.

Greater CGG repeat length was associated with reduced dominant hand ApEn in the 45% MVC condition (Figure 8A and Table 6; ρ = −0.529, *p* = 0.009). Greater CGG repeat length also was associated with increased dominant hand reaction time during the rapid task at 15% MVC (ρ = 0.543, *p* = 0.007). No other sensorimotor variables were associated with CGG repeat length.

**TABLE 6:** Correlational analyses of CGG and sensorimotor outcomes (Spearman ρ values).

		**2 s (“Rapid”)**			**8 s (“Sustained”)**		
		**MVC level**			**MVC level**		
	**Dependent variable**	**15%**	**45%**	**85%**	**15%**	**45%**	**85%**
Dominant hand	Rise phase reaction time	0.54^∗∗^	0.37	0.26	0.44^∗^	0.12	0.23
	Rate of force increase	0.10	0.14	0.15	–0.13	0.04	–0.10
	Rise phase duration	0.21	0.13	0.11	0.02	0.12	0.07
	Rise phase accuracy	–0.09	–0.08	–0.28	0.02	–0.05	–0.01
	
	ApEn	−	−	−	–0.33	−0.53^∗∗^	−0.45^∗^
	Force SD	−	−	−	0.22	0.19	0.32
	Mean force	−	−	−	–0.11	–0.13	–0.16
	
	Rate of force relaxation	–0.17	–0.01	–0.08	–0.27	0.12	0.14

Non-dominant Hand	Rise phase reaction time	0.30	0.38	0.40	0.46^∗^	0.36	0.45^∗^
	Rate of force increase	–0.09	0.02	–0.19	0.09	–0.22	–0.37
	Rise phase duration	0.30×10^−2^	0.06	0.15	0.39	0.35	0.34
	Rise phase accuracy	0.17	0.40	–0.07	0.29	0.27	–0.25
	
	ApEn	−	−	−	–0.39	−0.45^∗^	–0.38
	Force SD	−	−	−	0.35	0.18	0.08
	Mean force	−	−	−	–0.09	–0.10	–0.07
	
	Rate of force relaxation	0.11	0.07	0.22	–0.10	–0.17	0.24

*MVC: maximum voluntary contraction; CGG: cytosine-guanine-guanine; ^∗^*p* < 0.05; ^∗∗^*p* < 0.01. Rates of force relaxation are negative values, and so positive correlations indicate that an increase in CGG repeat length is associated with slower (i.e., less negative) rates of force relaxation.*

#### Clinical Symptoms

More severe FXTAS symptoms were associated with greater reaction times during the rapid task in the dominant hand 45% MVC condition (Figure 8B and Table 7; ρ = 0.700, *p* = 0.002), dominant hand 85% MVC condition (ρ = 0.665, *p* = 0.005), and non-dominant hand 85% MVC condition (ρ = 0.674, *p* = 0.003). More severe FXTAS symptoms also were associated with greater reaction times during the dominant hand 15% MVC condition of the sustained task (ρ = 0.612, *p* = 0.009). More severe ICARS scores were associated with higher force SD during the non-dominant hand 45% MVC condition (Figure 8C; ρ = 0.663, *p* = 0.004).

**TABLE 7:** Correlational analyses of total ICARS scores and sensorimotor outcomes (Spearman ρ values).

		**2 s (“Rapid”)**			**8 s (“Sustained”)**		
		
		**MVC level**			**MVC level**		
		
	**Dependent variable**	**15%**	**45%**	**85%**	**15%**	**45%**	**85%**
Dominant hand	Rise phase reaction time	0.52^∗^	0.70^∗∗^	0.67^∗∗^	0.61^∗∗^	0.18	0.34
	Rate of force increase	0.09	0.11	0.30	–0.09	–0.16	0.11
	Rise phase duration	–0.05	–0.11	–0.09	–0.21	0.07	0.09
	Rise phase accuracy	–0.21	–0.04	–0.23	–0.11	0.12	–0.42
	
	ApEn	−	−	−	–0.18	0.02	–0.20
	Force SD	−	−	−	0.22	0.40	0.41
	Mean force	−	−	−	0.14	0.08	0.09
	
	Rate of force relaxation	–0.39	–0.43	–0.44	–0.27	–0.16	–0.10
Non-dominant hand	Rise phase reaction time	0.46	0.52^∗^	0.67^∗∗^	0.37	0.44	0.25
	Rate of force increase	–0.11	–0.04	–0.07	–0.22	–0.05	–0.09
	Rise phase duration	–0.22	–0.13	–0.19	−0.20×10^−2^	0.13	−0.20×10^−2^
	Rise phase accuracy	–0.10	0.19	0.03	–0.04	0.22	–0.43
	
	ApEn	−	−	−	–0.40	–0.17	–0.38
	Force SD	−	−	−	0.34	0.66^∗∗^	0.58^∗^
	Mean force	−	−	−	0.09	0.14	0.11
	
	Rate of force relaxation	–0.34	–0.03	0.02	0.12	–0.01	0.38

*MVC: maximum voluntary contraction; ICARS: International Cooperative Ataxia Rating Scale; ^∗^*p* < 0.05; ^∗∗^*p* < 0.01. Rates of force relaxation are negative values, and so positive correlations indicate that an increase in CGG repeat length is associated with slower (i.e., less negative) rates of force relaxation.*

## Discussion

Despite sensorimotor impairments being central to the diagnosis of FXTAS, few studies have quantified precision sensorimotor behaviors in aging *FMR1* premutation carriers. Here, we examined multiple distinct component processes of precision sensorimotor behavior in aging premutation carriers in order to identify both spared and affected systems. Four key findings are documented. First, dominant hand strength was reduced relative to non-dominant hand strength in premutation carriers implicating atypical lateralized degeneration of neuromuscular systems in aging carriers of *FMR1* premutation alleles. Second, aging premutation carriers demonstrated a reduced ability to rapidly increase force during precision gripping suggesting alterations in feedforward sensorimotor control systems. Third, younger premutation carriers demonstrated reduced complexity of their sustained force output (i.e., ApEn), suggesting the ability to dynamically adjust motor output in response to sensory feedback may be impacted, especially during initial stages of aging during which premutation carriers first become vulnerable to FXTAS-associated deterioration. Last, multiple impairments of sensorimotor behavior were associated with CGG repeat length and clinically rated neuromotor issues in premutation carriers indicating that select precision measures of sensorimotor behavior may covary with FXTAS risk or progression.

### Reduced MVC in Aging *FMR1* Premutation Allele Carriers

Although premutation carriers and healthy controls did not differ on overall strength (i.e., MVC) or mean force output, premutation carriers showed a greater difference between their dominant and non-dominant hand MVC and mean force relative to controls. It is possible that premutation carriers show degeneration of neuromuscular systems as suggested by previous findings documenting reduced motor unit firing rates (Park et al., 2019). Findings that MVC reductions in premutation carriers may be more prominent in the dominant relative to the non-dominant hand suggest that neuromotor deterioration may be lateralized initially during aging or during initial stages of FXTAS. Few studies have examined lateralization of sensorimotor behavior in aging FMR1 premutation carriers or patients with FXTAS, but longitudinal studies tracking neuromuscular strength across both dominant and non-dominant hands are warranted.

### Rapid Force Production in Aging *FMR1* Premutation Allele Carriers

Reduced rates and increased durations of initial force output in aging premutation carriers together suggest impairment in the ability to rapidly increase force during precision sensorimotor actions. These findings likely are not attributable to diminished overall force output as we controlled for the overall amount of individuals’ force generation. Instead, premutation carriers appear to have a reduced ability to rapidly generate force, suggesting that the bradykinesia associated with FXTAS (Niu et al., 2014) may be evident in some asymptomatic aging premutation carriers during actions that require rapid increases in force. Similar reductions in initial force production also have been reported in studies of Parkinson’s disease suggesting basal ganglia circuit functions may be affected during aging in *FMR1* premutation carriers (Stelmach and Worringham, 1988; Fellows et al., 1998). This hypothesis is supported by studies highlighting increased iron deposition in neuronal and glial cells in putamen nuclei of FXTAS patients (Ariza et al., 2017) and case studies documenting pre- and post-synaptic nigrostriatal dysfunction (Zuhlke et al., 2004; Scaglione et al., 2008; Healy et al., 2009). Our findings also could reflect peripheral alterations. As suggested by our findings of increased lateralization of MVC in premutation carriers, atypical recruitment of motor neurons during voluntary muscle contractions is possible (Rose and McGill, 2005; Wang et al., 2017; Park et al., 2019). For example, a previous study has documented slower nerve conduction velocities and F-wave latencies in male premutation carriers with and without FXTAS (Soontarapornchai et al., 2008). EMG abnormalities, including reduced motor unit firing rates, have been reported in premutation carriers and FXTAS patients, indicating that difficulties generating force also may stem from alterations at the neuromuscular level including reduced rates of motor unit recruitment (Lechpammer et al., 2017; Bravo et al., 2018; Park et al., 2019).

### Sustained Sensorimotor Control in Aging *FMR1* Premutation Allele Carriers

During sustained force contractions, *FMR1* premutation carriers showed lower time series complexity (reduced ApEn), especially at lower force levels and at younger ages, reflecting a reduced ability to dynamically adjust force output in response to sensory feedback. Increased complexity of force output is adaptive and reflects individuals’ ability to integrate multiple sensory feedback and feedforward processes and update internal action representations that guide the precision of sensorimotor output during sustained behavior. Lower complexity suggests reduced integration of these distinct processes and reduced ability to update precision sensorimotor behavior to meet task demands. Our finding that the severity of ApEn reductions in premutation carriers is relatively similar in magnitude across ages for the non-dominant hand, but more prominent at younger ages for the dominant hand indicates that deterioration of sustained sensorimotor behavior may be lateralized in aging premutation carriers. More specifically, our results suggest that healthy controls show worsening of their sustained force control as they age, whereas the opposite pattern is true for premutation carriers when using the dominant hand. We postulate that older premutation carriers in our sample who currently report being asymptomatic may be less affected by aging effects of *FMR1* premutation alleles and less likely to develop FXTAS than the younger individuals in our sample who are beginning to age into the period of adulthood during which they are most likely to develop FXTAS symptoms. This hypothesis is supported by evidence that FXTAS prevalence decreases during late adulthood reflecting increased FXTAS-related mortality rates and reduced likelihood of FXTAS onset during elderly years (Rodriguez-Revenga et al., 2009). Our finding that reduced force complexity in premutation carriers is more severe at lower force levels indicates that deficits in sustained sensorimotor behaviors likely impact multiple tasks of daily living (e.g., lifting a glass of water) but may not manifest during more strenuous activities involving higher levels of isometric force.

Reduced complexity of the time-dependent structure of force oscillations in younger premutation carriers may reflect a reduced number of neural oscillators (Vaillancourt et al., 2001b). Neural oscillators within the central nervous system each generate rhythmic output. Corticomotor neurons demonstrate preferred discharge frequencies, and so the use of a larger number of neural oscillators to generate motor output would result in greater complexity of motor output as each neural oscillator contributes output of a different frequency (McAuley and Marsden, 2000). Likewise, fewer neural oscillators generating motor output would result in the reduced variability of motor output timing consistent with a less complex and more rhythmic force output (McAuley and Marsden, 2000; Vaillancourt et al., 2001b). Our findings of reduced ApEn in younger premutation carriers thus implicate atypical integration of neural oscillators that may contribute to increased rates of tremor (Homberg et al., 1987). ApEn measurements during sustained sensorimotor behavior hold promise for determining mechanisms contributing to tremor in FXTAS, and as surrogate biomarkers useful for clinical trials targeting tremor in patients (Hagerman et al., 2012). These findings also may be consistent with our recent study documenting greater sustained force variability in aging *FMR1* premutation carriers during finger abduction (Park et al., 2019). While we did not find evidence for atypical variability in premutation carriers in the present study, we did find that greater force variability was associated with more severe FXTAS symptoms suggesting that sustained sensorimotor dysmetria may be present in aging premutation carriers who are showing or beginning to show disease-related clinical issues. Ultimately, due to the relatively small effect sizes of ApEn interactions, it will be important to systematically assess sustained sensorimotor control targeting premutation carriers at the younger age range of our sample (i.e., 45–60 years) and in relation to FXTAS symptoms over time to determine the power of our objective measures of sensorimotor behavior to track FXTAS progression and risk.

### Sensorimotor Behavior and FXTAS Symptoms

In addition to identifying multiple sensorimotor behavioral alterations in aging *FMR1* premutation carriers, we also document multiple relationships between sensorimotor behavior and clinical symptoms of FXTAS. We found that increased reaction time and increased force variability each were associated with more severe clinically rated neuromotor issues in premutation carriers suggesting that quantifiable deficits in precision sensorimotor behaviors may be part of the aging process in *FMR1* premutation carriers, or that these issues may reflect early indicators of neurodegeneration associated with FXTAS. Our findings that slower reaction times across multiple task conditions (e.g., target force level, task length, hand) are associated with more severe clinical symptoms provide evidence that initial motor preparation and planning processes may deteriorate as part of the progression of FXTAS. Degeneration of premotor responses in individuals showing clinical signs of FXTAS may result from degeneration of motor fiber tracts that limits rapid processing of sensory information and generation of action plans (Greco et al., 2006; Wang et al., 2013). Our finding that greater force variability is associated with more severe FXTAS symptoms in premutation carriers indicates that a reduced ability to precisely maintain a steady motor output in response to sensory feedback information may track with developing symptoms in premutation carriers. Increased sustained force variability also is consistent with known neuropathological indicators of FXTAS. As individuals sustain a constant level of force using visual feedback, visual input is translated into motor corrections through parietal-ponto-cerebellar pathways. The MCP serves as the primary white matter input pathway relaying parietal-ponto visual feedback information to cerebellar circuits that encode reactive motor corrections to cortex (Stein and Glickstein, 1992). Degeneration of the MCP, reflected as hyperintensities on T2-weighted scans, is symptomatic of FXTAS and may contribute to both greater sensorimotor variability and FXTAS clinical symptoms (Jacquemont et al., 2003).

Based on prior studies showing that greater CGG repeat length among premutation carriers increases risk for FXTAS (Tassone et al., 2007), our finding that reduced ApEn was related to increased CGG repeat length in premutation carriers also suggests that sustained sensorimotor behavioral issues may covary with disease risk. From a more mechanistic perspective, greater CGG repeat length in the premutation range contributes to increased mRNA transcript, sequestration of proteins, and intranuclear inclusions (Greco et al., 2006; Li and Jin, 2012). These inclusions have been documented in pontine and cerebellar cells in the majority of cases studied to date (Greco et al., 2006; Ariza et al., 2016), suggesting that greater CGG repeat length compromises ponto-cerebellar functions. The atypical sensorimotor behaviors identified in this study are consistent with this model and may serve as objective biobehavioral targets useful for understanding pathophysiological processes associated with FXTAS and quantifying clinically relevant changes in aging premutation carriers.

### Limitations

Several limitations of the present study should be acknowledged. First, larger samples of FXTAS patients and asymptomatic premutation carriers are needed to examine variability in sensorimotor behavior during aging and determine disease-specific markers. Longitudinal samples are needed to track disease onset and progression and clarify the extent to which objective measures of sensorimotor precision may track with disease course. Second, it will be important to include movement disorder comparison groups in future studies of aging premutation carriers to determine the specificity of our sensorimotor markers to *FMR1* premutation carriers, though we propose that the next critical step is to determine the specificity of key sensorimotor issues to symptomatic compared to asymptomatic *FMR1* premutation carriers so that disease presence can be reliably identified in aging individuals who test positive for premutation alleles. Third, our sample consisted primarily of females who are at reduced risk for FXTAS relative to males. Despite 75% of our sample being female, we established multiple sensorimotor issues in aging premutation carriers and identified multiple participants, both male and female, showing FXTAS symptoms. Inclusion of females in FXTAS studies is warranted, though larger samples that allow for direct comparisons of sensorimotor behavior in aging males and females are needed. Fourth, while we report behavioral findings in relation to CGG repeat length, measures of mRNA, methylation ratios, and FMR protein are important for clarifying how aberrant neurobiological processes contribute to FXTAS risk or prodromal symptoms. Last, as with many hypothesis generating studies, the relatively small effect sizes of some of our group interactions highlight the need for replication. Still, our findings identify multiple sensorimotor targets and highlight important task conditions and demographic features that can be focused upon (e.g., increased sampling of middle-aged carriers) to characterize neurodegenerative processes associated with *FMR1* premutation alleles and FXTAS.

## Conclusion

Our results identify multiple precision sensorimotor issues in aging *FMR1* premutation carriers and indicate that select sensorimotor alterations track with FXTAS symptom severity. Together, these findings suggest that subclinical deficits of precision sensorimotor behavior may be detectable prior to the onset of FXTAS and serve as objective targets for tracking disease risk and monitoring disease progression.

## Data Availability Statement

The raw data supporting the conclusions of this manuscript will be made available by the authors, without undue reservation, to any qualified researcher.

## Ethics Statement

This study was carried out in accordance with the recommendations of the University of Texas Southwestern Medical Center Institutional Review Board with written informed consent from all subjects. All subjects gave written informed consent in accordance with the Declaration of Helsinki. The protocol was approved by the University of Texas Southwestern Medical Center Institutional Review Board.

## Author Contributions

MM was responsible for the conception and design of the study. PK and SW performed the clinical evaluations for *FMR1* premutation carriers. SL provided the radiological evaluations of the T2 scan images for premutation carriers. MM, ZW, and SW collected the behavioral data. ZW wrote the MATLAB scoring programs for the data analyses. MM, WM, and SK scored the raw data, performed the statistical analyses, and interpreted the results. WM and MM drafted and edited the manuscript. All authors approved the final version of the manuscript.

## Conflict of Interest

The authors declare that the research was conducted in the absence of any commercial or financial relationships that could be construed as a potential conflict of interest.
